# Ectopic Expression of Poplar *PsnCYCD1;1* Reduces Cell Size and Regulates Flower Organ Development in *Nicotiana tabacum*

**DOI:** 10.3389/fpls.2022.868731

**Published:** 2022-04-07

**Authors:** Zhongnan Zhao, Tangchun Zheng, Lijuan Dai, Yi Liu, Shuang Li, Guanzheng Qu

**Affiliations:** ^1^State Key Laboratory of Tree Genetics and Breeding, Northeast Forestry University, Harbin, China; ^2^National Engineering Research Center for Floriculture, School of Landscape Architecture, Beijing Forestry University, Beijing, China

**Keywords:** *Populus*, *PsnCYCD1;1*, cyclin, small cell, enlarged floral organs

## Abstract

The D-type cyclin (CYCD) gene, as the rate-limiting enzyme in the G1 phase of cell cycle, plays a vital role in the process of plant growth and development. Early studies on plant cyclin mostly focused on herbs, such as *Arabidopsis thaliana*. The sustainable growth ability of woody plants is a unique characteristic in the study of plant cyclin. Here, the promoter of *PsnCYCD1;1* was cloned from poplar by PCR and genetically transformed into tobacco. A strong GUS activity was observed in the areas with vigorous cell division, such as stem tips, lateral buds, and young leaves. The *PsnCYCD1;1-GFP* fusion expression vector was transformed into tobacco, and the green fluorescence signal was observed in the nucleus. Compared with the control plant, the transgenic tobacco showed significant changes in the flower organs, such as enlargement of sepals, petals, and fruits. Furthermore, the stems of transgenic plants were slightly curved at each stem node, the leaves were curled on the adaxial side, and the fruits were seriously aborted after artificial pollination. Microscopic observation showed that the epidermal cells of petals, leaves, and seed coats of transgenic plants became smaller. The transcriptional levels of endogenous genes, such as *NtCYCDs*, *NtSTM*, *NtKNAT1*, and *NtASs*, were upregulated by *PsnCYCD1;1*. Therefore, *PsnCYCD1;1* gene played an important role in the regulation of flower organ and stem development, providing new understanding for the functional characterization of *CYCD* gene and new resources for improving the ornamental value of horticultural plants.

## Introduction

Plant development is accompanied by cell division, which is regulated by cell cycle activity (Inzé and De Veylder, 2006). Plant D-type cyclins control the process of cell cycle and play an important role in cell division and proliferation. D-type cyclins are preferentially induced by mitogen stimulants in the G1 phase to accumulate developmental signals (Oakenfull et al., 2002; Richard et al., 2002) and control cells to re-enter the cell cycle (Soni et al., 1995; Meijer and Murray, 2001). D-type cyclins (CYCDs) are called G phase-specific cyclins due to their important role in the transition from G1 to S phase (De Veylder et al., 1999).

Ten genes encoding CYCDs have been identified in *Arabidopsis*. They are divided into seven subfamilies (CYCD1-CYCD7), of which CYCD3 subfamily has three members, CYCD4 subfamily has two members, and the other five CYCD subfamilies are encoded by only one gene (Vandepoele et al., 2002). *CYCD1;1*, *CYCD2;1*, and *CYCD3;1* are originally screened from *Arabidopsis*, which can restore the phenotype of yeast G1 cyclin mutant (Soni et al., 1995). Cyclins, as a regulatory subunit of protein kinase, closely bind to CDK, forming an active kinase complex, and directionally regulate plant growth and development (Morgan, 1995). Most CYCD proteins can interact with cyclin-dependent kinase A;1 (CDKA;1), and the overexpression of some *CYCD* genes promote the cells to enter the S phase (Masubelele et al., 2005; Menges et al., 2006; Mendez et al., 2020), indicating that the CDKA-CYCD complex regulates the conversion of G1/S phase.

Many studies have shown that overexpression of some *CYCDs* in plants can change plant growth and development. The constitutive expression of *CYCD* enables transgenic *Arabidopsis* leaves to undergo cell division without adding exogenous cytokinin (Riou-Khamlichi et al., 2000). The *Populus trichocarpa PtCYCB1* promoter is used to drive the expression of the *CYCD* gene in tobacco, which improves the cell division of vascular cambium and increases the differentiation of secondary xylem in tobacco (Fujii et al., 2012). The ectopic expression of *AtCYCD2* accelerates the growth and aboveground biomass accumulation rate of transgenic tobacco from the seedling stage to maturity (Boucheron et al., 2005; Kopertekh and Reichardt, 2021). Moreover, overexpressing the *CYCD3* gene into tobacco also accelerates the growth rate of leaves and changes the structure of the shoot apical meristem (Boucheron et al., 2005). The *CYCD3;1* transgenic *Arabidopsis* develops slowly, but the flowering time does not change. Meanwhile, overexpression of *CYCD3;1* also causes changes in leaf morphology and structure, particularly, increased number of leaf epidermal cells and curled leaves toward the medial axis (Dewitte and Murray, 2003). Soybean D-type cyclin (*GmCYCD6;1-6*) forms a feedforward loop which is the key mechanism to regulate the initial division of soybean nodule primordium (Wang et al., 2022). The seed formation is controlled by the activity of CYCD7 in the central cell and in the developing endosperm (Sornay et al., 2015).

Previous reports on CYCDs were not comprehensive and mostly focused on genes, such as CYCD2, CYCD3, and CYCD7, from model plants and herbs. There are few reports on the function of cyclin genes in woody plants. We cloned a *CYCD1;1* from poplar and performed a preliminary analysis in *Arabidopsis* (Zheng et al., 2021). Significant phenotypic changes were observed in transgenic tobacco by constructing plant expression vector, and these phenotypes were not found in previous transgenic *Arabidopsis*. Therefore, this study not only improves the function of *PsnCYCD1;1*, but also enriches the new understanding of plant cyclin.

## Materials and Methods

### Plant Material and Growth Conditions

The *Populus simonii* × *P. nigra* cross was obtained from the campus of Northeast Forestry University in Heilongjiang Province, China. The young leaves were sampled, immediately frozen in liquid nitrogen, and stored at −80°C before the extraction of genomic DNA. *Nicotiana tabacum* L. was planted in a pot containing cultivation substrate (turf peat and pearlite, 2:1 v/v) under controlled conditions with 16 h of light/8 h of darkness, 22 ± 2°C temperature, 40–50% humidity, and 200 μmol m^–2^ s^–1^ light intensity.

### Cloning of *PsnCYCD1;1* Gene

To verify the stable subcellular localization of *PsnCYCD1;1* gene, PCR cloning was carried out with the specific primers (Supplementary Table 1) by removing the stop codon and using pUC18-*PsnCYCD1;1* plasmid as a template (Zheng et al., 2021). The reaction system consisted of 2.5 μl 10 × Ex PCR buffer, 2.0 μl dNTP (10 mmol/L), and 1.0 μl upstream and downstream primers, 0.25 μl *ExTaq* (5 U/μl), 0.1 μl plasmid template (10 ng/μl), and added ddH_2_O to make final volume of 25 μl. The amplification procedure was as follows: 35 cycles at 94°C for 4 min, 94°C for 30 s, 56°C for 30 s, and 72°C for 1.5 min, and 72°C for 7 min. After the reaction, 5 μl of PCR products were detected in 1% agarose gel. Then, the PCR product gel was recovered, connected to the pEasy-T1 vector, transformed into *Escherichia coli* Trans1-T1 competent cells, and screened for positive clones.

### Construction of Plant Expression Vector

Restriction endonucleases *Xba*I and *Kpn*I were used to digest pEasy-*PsnCYCD1;1* and pROKII-*GFP* vectors, respectively. The enzyme reaction system consisted of 1.0 μl *Xba*I, 1.0 μl *Kpn*I, 2.0 μl 10× K buffer, 1 μg plasmid, and ddH_2_O to make the final volume of 20 μl. After the target fragment was recovered by gel and linked by T_4_ DNA ligase, the recombinants were transformed into *E. coli* Trans1-T1 competent cells and positive colonies were screened for sequencing verification. Finally, the pROKII-*PsnCYCD1;1-GFP* plasmid was transferred into *Agrobacterium* GV3101 competent cells using liquid nitrogen freezing and thawing method (Chen et al., 1994).

### Cloning of *PsnCYCD1;1* Promoter and Construction of Plant Expression Vector

The DNA of young leaves of *P. simonii* × *P. nigra* was isolated by hexadecyltrimethylammonium bromide (CTAB) method (Doyle, 1987), and was used as a template to amplify promoter fragment by specific primers that were designed based on the upstream 1,800 bp sequences of *PsnCYCD1;1*. To verify spatiotemporal expression pattern of *PsnCYCD1;1* promoter, after cloning and purification of *PsnCYCD1;1* promoter fragment, the target fragment, and pBI121-*GUS* were digested by *Hin*dIII and *Kpn*I and linked by T_4_ DNA ligase to construct the target vector pBI121*-PsnCYCD1;1pro*-*GUS*. After verification by sequencing, the recombinant plasmid was transferred into *Agrobacterium* GV3101 competent cells using the liquid nitrogen freezing and thawing method.

### Genetic Transformation

Recombinants (pROKII-*PsnCYCD1;1-GFP* and pBI121*-PsnCYCD1;1pro*-*GUS*) were transferred into wild-type tobacco by leaf disc method (Zheng et al., 2014). The final resistant plants were obtained through differentiation on a selective medium containing 50 mg/L kanamycin. After stem growth and rooting culture, the positive plantlets were used to extract DNA from the leaves by the CTAB method. PCR was performed using vector primers, and the products were evaluated by 1% agarose gel electrophoresis.

### Quantitative Real-Time PCR Analysis

To validate the transcriptional level of the *PsnCYCD1;1* gene, the total RNA of young leaves of transgenic plants was extracted using a MiniBEST Plant RNA Extraction Kit (TaKaRa, Beijing, China). Total RNA was reverse transcribed into cDNA and diluted 10 times with ddH_2_O as a template. The reaction system was 20 μl, containing 10 μl SYBR Green, 0.4 μl Rox dye II, 2 μl cDNA template, and 0.8 μl forward and reverse primers, respectively (Supplementary Table 1). The reaction procedure was as follows: 40 cycles at 95°C for 30 s; 95°C for 5 s, 60°C for 35 s; 95°C 15 s, 60°C 1 min, and 95°C 15 s. The *Ntactin* gene was used as an internal reference (Zheng et al., 2014). Three biological and technical repetitions were performed, and the relative expression of the target gene was calculated with the 2-delt cycle threshold (CT) method (Livak and Schmittgen, 2001).

To detect the transcriptional changes of endogenous gene-related stem and leaf development, the total RNA of young stems and leaves of 3-week-old control and transgenic tobacco was isolated. Fourteen gene-specific primers were designed using Integrated DNA Technologies (IDT) online tools^1^ (Supplementary Table 1). Housekeeping gene and reaction system were the same as above.

### Subcellular Localization

The root tips of 2-week-old transgenic tobacco (pROKII*-PsnCYCD1;1-GFP*) seedlings were collected and flatten on a glass slide for microscopic observation. The wild-type and transgenic tobacco with a pROKII-*GFP* vector was used as control. The green fluorescent signals in cells were observed and photographed by Leica TCS SP8 laser confocal microscope (Leica, Wetzlar, Germany).

### GUS Staining

Transgenic tobacco (pBI121-*PsnCYCD1;1pro*-*GUS*) seedlings and different tissues in different growth stages were immersed in β-glucuronidase (GUS) staining solution, and the interstitial gas of tissues were discharged with a vacuum pump to maintain the air pressure at 0.01 Pa for about 30 min. Subsequently, the tissues were dyed in a 37°C incubator for 5–7 h and fixed overnight with Carnot fixative, soaked in 75% alcohol for 2–3 days until completely faded, and photographed with Leica ED4 stereomicroscope (Leica, Germany).

### Phenotypic Observation of Transgenic Tobacco

Wild-type and three transgenic lines (pROKII*-PsnCYCD1;1-GFP*) with high expression levels were planted in pots in a greenhouse with controlled environmental conditions. Leaf development and stem growth were recorded every 2 days. Five newly blooming flowers were randomly selected from each plant, and the width of the corolla, the length of petal, calyx, fruit, and seed were measured and statistically analyzed.

To observe the seed development, the transgenic and control plants planted under the same growth conditions were selected. After 3 days, when the flowers withered and the fruit was developing, the peel was peeled off and the developing seeds were observed and photographed under the microscope. One week later, the abortion of developing seeds was statistically analyzed.

### Environmental Scanning Electron Microscope Observation

Dry seeds were sprayed with gold powder before scanning and observation. Fresh samples of petals and leaves were cut into sheets less than 1 cm wide and 1.5 cm long before being directly placed on the objective table of environmental scanning electron microscope (ESEM) for observation and photography with Philips Quanta 200 (FEI, Eindhoven, Netherlands). The environmental vacuum mode was selected for the electron microscope sample chamber, and the gas secondary electronic signal imaging was adopted. The working conditions were as follows: high voltage at 12.5 KV, pressure >7.5e−3 Torr, filament current of 2.34 A, and emission current at 97 M a.

The relative size of epidermal cell and seed between transgenic tobacco and controls were measured and statistically analyzed by ImageJ software (National Institutes of Health, Bethesda, MD, United States).

## Results

### Cloning and Genetic Transformation of *PsnCYCD1;1* Gene and Promoter in Tobacco

A specific band of about 1,000 bp was amplified from pUC18-*PsnCYCD1;1* (Supplementary Figure 1), and the *PsnCYCD1;1* gene was confirmed by sequencing. Then, the recombinant pROKII-*PsnCYCD1;1-GFP* was obtained by connecting *PsnCYCD1;1* and pROKII-*GFP* by double enzyme digestion. A 1,941 bp nucleotide sequence of the upstream of *PsnCYCD1;1* gene was cloned using leaf DNA from *P. simonii* × *P. nigra* as template (Supplementary Figure 2), while the recombinant pBI121*-PsnCYCD1;1pro*-*GUS* was obtained by inserting into pBI121-*GUS* with restriction endonuclease. Sequencing results showed that the target fragment was an upstream sequence of the *PsnCYCD1;1* gene and the *cis*-elements of *PsnCYCD1;1* promoter were analyzed using PlantCARE (Supplementary Figure 3).

The genetic transformation was carried out by leaf disc method with *Agrobacterium* GV3101 containing pROKII-*PsnCYCD1;1-GFP* and pBI121*-PsnCYCD1;1pro*-*GUS* vectors, respectively. Then, 19 independent *35S:PsnCYCD1;1-GFP* transgenic tobacco and 16 independent *PsnCYCD1;1pro*-*GUS* transgenic tobacco T_1_ lines were obtained by DNA PCR, respectively. The positive plants were further detected by quantitative real-time PCR (qRT-PCR) to detect the relative expression level of *PsnCYCD1;1* (Supplementary Figure 4). Finally, nine independent lines in the homologous T_2_ generation were screened, and four lines (OE1, OE2, OE6, and OE14) were further chosen to analyze the phenotypic changes.

### GFP Observation of Transgenic Tobacco Root Tips

**FIGURE 1:**
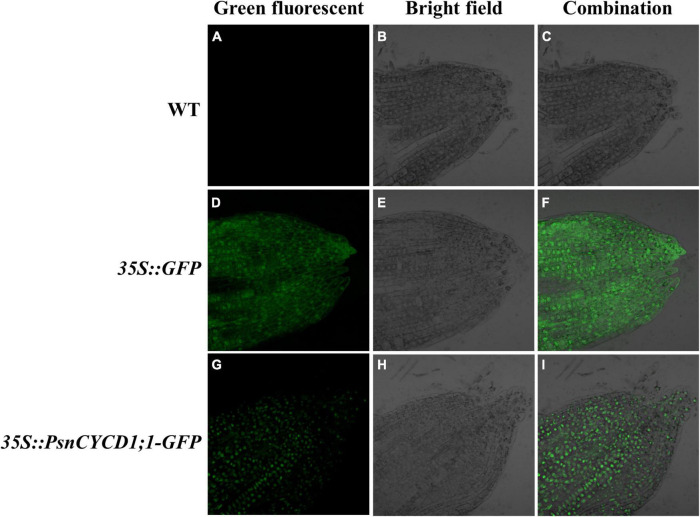
Subcellular location of green fluorescent protein (GFP) and PsnCYCD1;1-GFP protein in tobacco root tip. Green fluorescent **(A,D,G)**: green fluorescence signal; bright field **(B,E,H)**: white light; and combination **(C,F,I)**: combined signals of different fluorescence. WT, wild-type; *35::GFP*, pROKII-*GFP*, empty vector control; *35S::PsnCYCD1;1-GFP*, fusion vector of pROKII-*PsnCYCD1;1-GFP*.

To validate the subcellular localization of *PsnCYCD1;1* in plants, the root tips of T_2_ generation seedling of transgenic (*35S:PsnCYCD1;1-GFP*), empty vector (*35:GFP*), and wild-type tobacco were compressed and observed under laser confocal microscope. The results showed that in the root tips of wild-type tobacco, there was no fluorescent signal. In empty vector control (CK) and tobacco root tip cells, the green fluorescence signal was detected in the whole cell, while in *35S:PsnCYCD1;1-GFP* transgenic tobacco root tip cells, the green fluorescence signal was only detected in the nucleus, indicating that PsnCYCD1;1 performs biological functions in the nucleus (Figure 1).

### GUS Activity of *PsnCYCD1;1pro-GUS* Transgenic Tobacco

**FIGURE 2:**
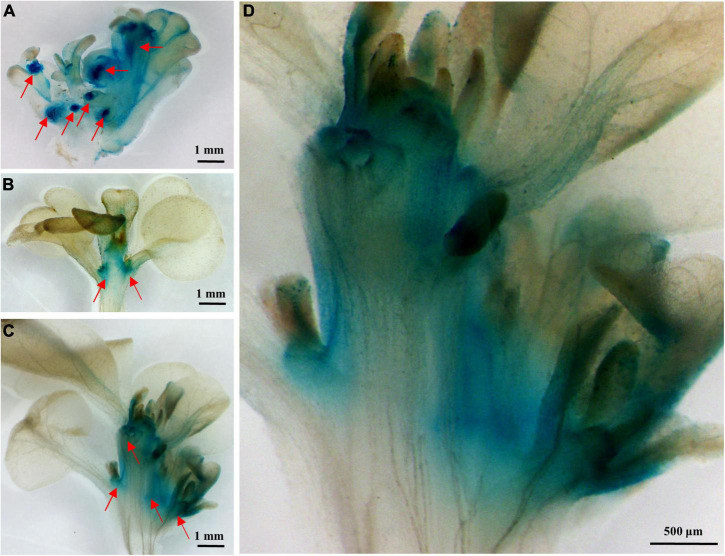
Histochemical analysis of β-glucuronidase (GUS) activity of *PsnCYCD1;1pro-GUS* transgenic tobacco. **(A)** Adventitious buds; **(B)** stem tip; **(C)** axillary bud development of stem tip; and **(D)** partial enlargement of **(C)**.

To study the function of the *PsnCYCD1;1* promoter, the homozygous T_2_ generation was screened and observed by histochemical staining. The results showed deep GUS staining at the growth point, terminal bud, and leaf bud. The coloring was very light and almost invisible in other parts, such as in leaves or stem segments, suggesting that the strong expression of *PsnCYCD1;1* in the meristem (Figure 2).

### Morphological Phenotype of Tobacco Overexpressing *PsnCYCD1;1*

**FIGURE 3:**
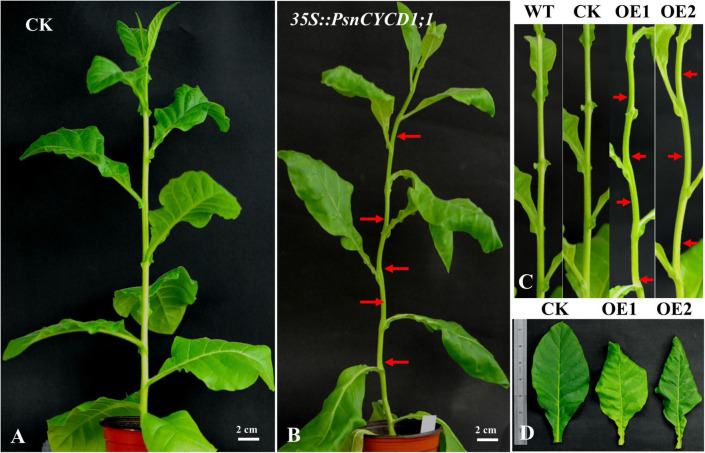
Phenotype observation of empty vector control (CK) and *35S:PsnCYCD1;1-GFP* tobacco. **(A)** Three-month old CK tobacco; **(B)** 3-month old *35S:PsnCYCD1;1-GFP* transgenic tobacco; **(C)** stem morphology of transgenic, wild-type, and CK tobacco; and **(D)** comparison of leaf morphology between transgenic and CK tobacco; WT, wild-type; CK, empty vector control; OE1/2, two individual transgenic lines.

**FIGURE 4:**
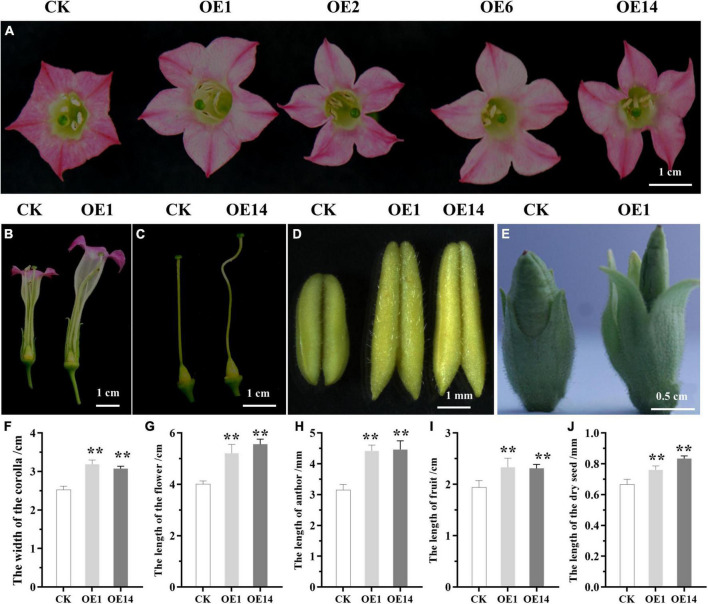
The differences in reproductive organs between CK and transgenic tobacco. **(A)** Comparison of the morphology of corollas; **(B)** longitudinal cutting of floral organs flowers; **(C)** comparison of the morphology of pistils; **(D)** comparison of the size of anthers; **(E)** comparison of the morphology of fruits; **(F–J)** statistical analysis of the phenotype of corolla, flower, anther, fruit, and dry seed. CK, empty vector control; OE1/2/6/14, four individual transgenic lines. Asterisks on the column diagram showed the significant difference between different samples (*p* ≤ 0.01).

In the vegetative growth stage, the leaves of transgenic tobacco were hypertrophic and could not be flattened normally compared with the control lines (Supplementary Figure 5 and Figure 3). Meanwhile, the stem of transgenic tobacco was not straight, showing irregular zigzag between two leaf nodes (Figure 3). After entering the reproductive growth stage, compared with the wild-type and CK lines, the transgenic lines had larger corolla, larger petals and sepals, and longer fruits and seeds. In addition, the length of stigma increased and showed a twisted phenotype (Figure 4). Three transgenic lines (OE1, OE2, and OE14) and three CK lines were selected, and five flowers were randomly taken from the top of each plant. The width of corolla and the length of sepal, petal, fruit, and seed were measured and analyzed by using SPSS 19.0 software and the *q* test. The results showed that compared with CK plants, the corolla of transgenic plants was significantly wider, the petals were significantly larger, and the sepals, fruits, and seeds were significantly longer (Figure 4), suggesting that ectopic expression of *PsnCYCD1;1* in tobacco affects the development of flower organs and fruits. Under normal conditions, the tobacco used in this study gradually withers after flowering and fruiting, and the whole growth cycle usually spans 3–5 months. However, some transgenic lines maintained long-term growth, and OE14 maintained flowering for more than 20 months, thus becoming a perennial plant with new individuals sprouting and growing from the axillary buds (Supplementary Figures 6, 7).

### Comparative Analysis of Fertility of Transgenic Tobacco

**FIGURE 5:**
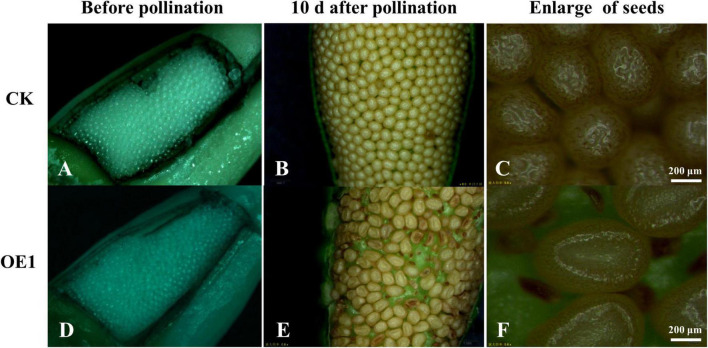
Ectopic expression of *PsnCYCD1;1* caused seed fertility in transgenic tobacco. **(A,D)** Observation of ovules before pollination between CK and transgenic (OE1) line; **(B,E)** microscopic observation of seeds in the ovary 10 days after pollination with wild-type pollens between CK and transgenic (OE1) line; and **(C,F)** close-up of the image **(B,E)**. CK, control plant **(A–C)**; OE1, transgenic plant **(D–F)**.

Although transgenic tobacco produces a large number of flowers, 100% of them fall off early and cannot develop into mature fruits. Through artificial pollination, few fruits can continue to develop, but the seed yield is very low (Supplementary Figure 8). Three transgenic lines and control lines were selected to observe seed development before pollination and 10 days after artificial pollination. After peeling, the unpollinated ovules and seeds were observed under the microscope and the abortion rate per unit area was calculated. The results of statistical analysis showed that the ovule development of control and transgenic plants was basically the same before pollination. After pollination with wild-type pollens, there were significant differences in fruit development between the CK and the transgenic line (*PsnCYCD1;1*), to which the abortion rates per unit area were 3.2 and 46.1%, respectively (Figure 5 and Supplementary Figures 8, 9), suggesting that the ectopic expression of *PsnCYCD1;1* can cause severe male sterility and slight female sterility in transgenic tobacco.

### Comparative Analysis of Cell Size of Transgenic Tobacco

**FIGURE 6:**
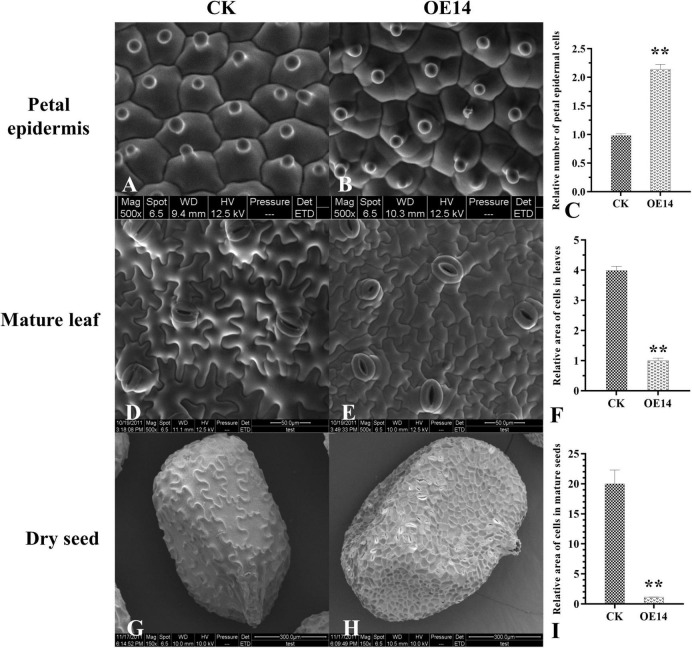
Comparative analysis of cell size between CK and transgenic (OE14) tobacco by environmental scanning electron microscope (ESEM). **(A,B)** ESEM observation of the petal epidermis between CK and transgenic (OE14) line. **(D,E)** ESEM observation of the abaxial epidermis of the leaves between CK and transgenic (OE14) line. **(G,H)** Microscopic observation of dry seeds between CK and transgenic (OE14) line. **(C,F,I)** Statistical analysis of the petal epidermis, mature leaf, and seed coat cells between CK and transgenic (OE14) lines. Asterisks on the column diagram showed the significant difference between different samples (*p* < 0.01).

To reveal the reasons for the enlargement of flower organs, the cell size of different tissues of transgenic plants was observed. Three fully bloomed petals from OE14 and CK tobacco were observed by ESEM. The results indicated that the normal papillary cells existed in the adaxial petal epidermis of CK tobacco (Figures 6A–C). Interestingly, there were many small cells formed by subsequent division of the perianth cells, and there were no papillary apexes on these small cells, suggesting that the ectopic expression of *PsnCYCD1;1* caused the morphological changes of perianth cells in transgenic tobacco.

The mature leaves of OE14 and CK tobacco with the same growth rates were selected, and the abaxial epidermis of the leaves was observed and photographed by ESEM. The results showed that the cell size of the abaxial epidermis cells of the OE14 was significantly different from that of the CK (Figures 6D,E). The relative cell size of the OE14 was only 25% of CK cells (Figure 6F).

To avoid the difference of growth period, the mature seeds were further selected for ESEM. The results showed that the surface of the seed coat of CK tobacco had reticular decoration (degenerated integument epidermis), in which the mesh outline was mostly irregular and in the form of round polygons with different sizes (Figure 6G). However, the net ridge of the reticular pattern on the seed coat surface of the OE14 seeds was thin, and the mesh outline was shaped like a regular polygon and looked like a honeycomb (Figure 6H). Statistical analysis of the size of a single cell on the seed epidermis showed that there was a great difference between the OE14 and the CK cells, and the relative sizes of OE14 cells were only about 1/20 of the CK cells (Figure 6I).

### Detection of Endogenous Related Genes in Transgenic Tobacco

**FIGURE 7:**
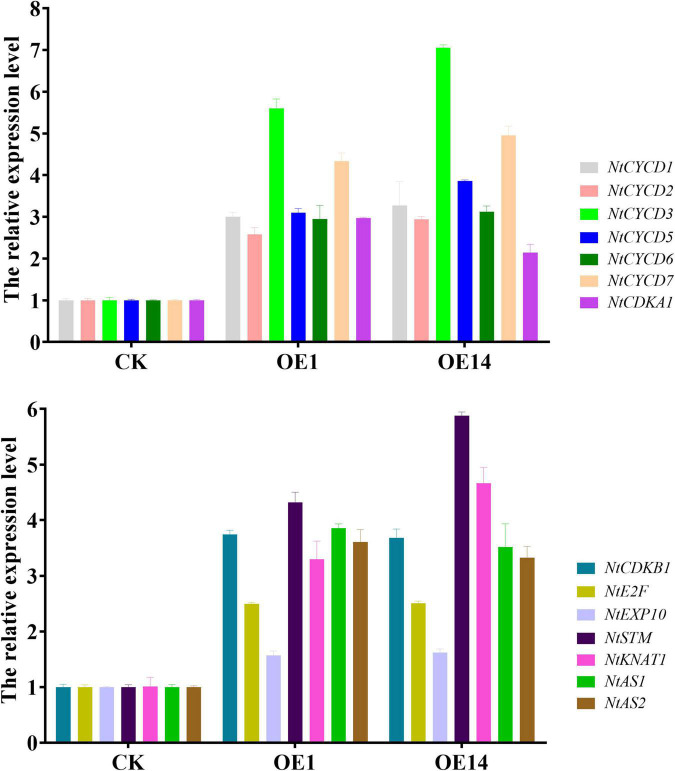
Quantitative real-time PCR analysis of endogenous related genes in CK and transgenic tobacco. CK, empty vector control tobacco; OE1/14, two individual transgenic lines.

To detect the transcriptional changes of other D type cyclins, cyclin kinases, downstream division genes, and stem development-related genes in *35S:PsnCYCD1;1-GFP* transgenic tobacco, the total RNA of young stems and young leaves of 3-week-old CK and transgenic (OE1 and OE14) seedlings was extracted, and the transcription levels of fourteen genes were detected by qRT-PCR. Compared with the CK, the endogenous *NtCYCD* genes were upregulated for more than two times in the transgenic plants. In particular, the expression of *NtCYCD3* gene was upregulated for about seven times. Cyclin kinases, *NtCDKA1* and *NtCDKB1*, and downstream division-related gene *NtE2F* were also upregulated more than two times in transgenic plants. In addition, stem development-related genes, *NtSTM* and *NtKNAT1*, and leaf polarity-related genes, *NtAS1* and *NtAS2*, were upregulated more than two times in transgenic plants (Figure 7).

## Discussion

The organs of higher plants continuously develop in the whole life cycle, which depends on the flexible control of cell division and cell proliferation. D-type cyclins are rate-limiting enzymes in the G1 phase, which determines the key period of cell differentiation. In *Arabidopsis*, ten genes encoding CYCDs have been identified and they are divided into seven subfamilies (CYCD1-CYCD7) (Vandepoele et al., 2002). In woody plant *Populus tomentosa*, 22 *PtrCYCD* genes are identified and divided into six subfamilies, including five *CYCD1* members, two *CYCD2/CYCD4* members, six *CYCD3* members, three *CYCD5* members, five *CYCD6* members, and one *CYCD7* gene (Dong et al., 2011). In view of the genome and secondary growth differences between woody plants and herbs, the number of cyclins in poplar is more than twice as compared to *Arabidopsis*, and some genes may have functional redundancy or special functions.

The subcellular localization results of PsnCYCD1;1 showed its location in the nucleus (Figure 1), which is consistent with the homologous genes located in the nucleus of *Arabidopsis*, such as AtCYCD 1;1, AtCYCD2;1, and AtCYCD3;1 (Planchais et al., 2004; Sanz et al., 2011). In transgenic tobacco, strong fluorescence signals were observed in root apical meristem (RAM) and lower epidermis of leaves (Supplementary Figure 10), suggesting that PsnCYCD1;1 is also a relatively stable protein, possibly due to the ectopic expression of *PsnCYCD1;1* gene in tobacco. Strong GUS activity was detected in the growth points, axillary buds, and young leaves, similar to the qRT-PCR results and confirming the high *PsnCYCD1;1* expression in the stem, flower, root, and young leaves (Zheng et al., 2021). The *cis*-element in *PsnCYCD1;1* promoter was predicted, and it was found that the *PsnCYCD1;1* promoter contained many tissue-specific expression elements (CAT-box, Skn-1_motif, and CCGTCC-box) and a variety of hormone response elements (ABRE and TGA elements), suggesting an important role of *PsnCYCD1;1* in poplar stem growth and lateral bud formation through hormone signaling pathways. In chrysanthemum, the expression level of *CYCD* was upregulated by cytokinin, auxin, and sugar together, causing a short internodal distance (Sun et al., 2021).

Plant growth and development are controlled in RAM and stem apical meristem (SAM). Meristem is the most active region of cell division. Therefore, the change in cell cycle may affect the downstream development process. Overexpression of *CYCDs* can change plant growth and development. Due to the reduction of G1 phase, the development process of *CYCD2* transgenic tobaccos is accelerated at all stages, from seed to adult plant growth, but the size of cells and meristem is normal (Cockcroft et al., 2000). However, overexpression of *CYCD2* in *Arabidopsis* showed no significant phenotypic changes (Cockcroft et al., 2000). In contrast, overexpression of *CYCD3* in *Arabidopsis* caused morphological changes, such as delayed SAM and leaf senescence and leaf explant tissues, proliferated independent of cytokinin (Riou-Khamlichi et al., 1999). Overexpression of *PtoCYCD3;3* in poplar promoted the development of cambium and vascular bundle (Guan et al., 2021). Targeted expression of *AtCYCD7;1* to the central cells and early endosperm reduced the seed number in each silique and increased seed size (Sornay et al., 2015, 2016). There are few reports on the overexpression of *CYCD1* gene in *Arabidopsis*. Ectopic expression of *PsnCYCD1;1* gene from poplar promoted cell division and produced curved rosette leaves in *Arabidopsis* (Zheng et al., 2021). Here, a new phenotype was found after the transfer of *PsnCYCD1;1* gene into tobacco. Compared with the control plant, the flower organs of transgenic tobacco overexpressing *PsnCYCD1;1* showed a significant increase in the width of corolla and the length of petals and sepals. At the same time, the seeds of transgenic tobacco had a high abortion. In addition, the cells of petals, lower epidermis of leaves, and seeds of transgenic plants became smaller, which was similar to the result of overexpression of *AtCYCD2;1* in *Arabidopsis*, causing significantly smaller cells in the root tip division area (Qi and John, 2007). Flower bud differentiation is a physiological and morphological sign of transformation from vegetative to reproductive growth. The differentiation order of each round of flower organs on the flower primordium is generally a centripetal differentiation. In this study, the calyx, petal, anther, and pistil were significantly larger, suggesting an important role of *PsnCYCD1;1* in heterologous regulation of cell cycle activities and plant reproductive development. Tobacco petal epidermis cells usually have papilla cells. We found comparatively smaller cells in the petal epidermis of transgenic plants, and some small cells did not have papillae. It is suggested that the overexpression of *PsnCYCD1;1* not only accelerates cell division, but also affects cell differentiation. Overexpression of *CYCD3;1* gene in *Arabidopsis* rendered the cells of transgenic plants smaller and less differentiated than wild-type cells. Meanwhile, there were no normal palisade and spongy tissue as these were replaced by a large number of smaller cells (Dewitte and Murray, 2003). Altogether, *PsnCYCD1;1* accelerates the process of mitosis in transgenic plants, resulting in a large number of cells. This may be due to an increased need for energy and nutrients for cytoplasmic growth in transgenic plants.

To investigate the reason for formation of small cells in transgenic tobacco, genes related to cell division and stem growth were identified, including six *NtCYCDs*, two *NtCDKs*, one *NtE2F*, one *NtEXP10*, one *NtSTM*, one *NtKNATs*, and two *NtASs*. The result of qRT-PCR showed that two D-type cyclins (*NtCYCD3* and *NtCYCD7*) were significantly upregulated (Figure 7), which is similar to the report of homologous genes. For example, *AtCYCD3;3* is involved in the lateral bud and root tip differentiation of *Arabidopsis* (Gaudin et al., 2000), while *AtCYCD7* regulates the development of embryo and root tip (Sornay et al., 2015). The *CDKB* is specific in plants (Boudolf et al., 2001), and CDKB2-GUS fusion protein showed intermittent expression patterns in *Arabidopsis* meristem (Adachi et al., 2006). The result of qRT-PCR showed that *NtCDKA1* and *NtCDKB1* were significantly upregulated, which is consistent with the report that CYCD and CDKA can form complexes (Mendez et al., 2020). The change of *E2F* expression can cause cell division and elongation of RAM and change the expression of some cell cycle regulatory factors, including *E2Fa*, *E2Fb*, and *E2Fe/DEL1* (Sozzani et al., 2009). The result of qRT-PCR showed that the expression of downstream division gene *NtE2F* was significantly upregulated, suggesting that the interaction among *E2Fs* may control the cell proliferation during organ formation and tissue development. Stem and leaf development-related genes (*NtSTM*, *NtKNAT1*, *NtAS1*, and *NtAS2*) were also upregulated in transgenic lines. *AS* gene regulates the paraxial and abaxial polarity development in leaves (Rast and Simon, 2012). The loss of the *ANT* gene function leads to the formation of small organs, while its overexpression increases the size of organs (Mizukami and Fischer, 2000). *KNAT*, mainly expressed in apical meristem, is inhibited by auxin and *AS* gene, and it participates in meristem regulation by cytokinin and *CYCD3* together with *STM* gene (Scofield et al., 2013).

In this study, the heterologous expression in tobacco may not always reflect the endogenous biological function in native species. Obtaining transgenic poplars to verify the phenotypic changes and evaluating the role of *cis*-elements in the regulation of flower organ and stem development is recommended to be the main objective in the future.

## Data Availability Statement

The original contributions presented in the study are included in the article/Supplementary Material, further inquiries can be directed to the corresponding authors.

## Author Contributions

ZZ, TZ, and GQ conceived and drafted the manuscript. ZZ and TZ performed the experiment. ZZ, TZ, LD, YL, and SL analyzed the data. GQ provided plant resources. TZ and GQ contributed to the conception of the study and finalized the manuscript. All authors read and approved the final manuscript.

## Conflict of Interest

The authors declare that the research was conducted in the absence of any commercial or financial relationships that could be construed as a potential conflict of interest.

## Publisher’s Note

All claims expressed in this article are solely those of the authors and do not necessarily represent those of their affiliated organizations, or those of the publisher, the editors and the reviewers. Any product that may be evaluated in this article, or claim that may be made by its manufacturer, is not guaranteed or endorsed by the publisher.

## Footnotes

1
https://sg.idtdna.com/pages
