# Supplementary material for: Ectopic Expression of Poplar PsnCYCD1;1 Reduces Cell Size and Regulates Flower Organ Development in Nicotiana tabacum
Source: Front Plant Sci. 2022 Apr 7;13:868731. doi: 10.3389/fpls.2022.868731 (PMC9021869; doi:10.3389/fpls.2022.868731)
Supplement: Supplementary file 1 [file Data_Sheet_1.PDF]

Supplementary Figures

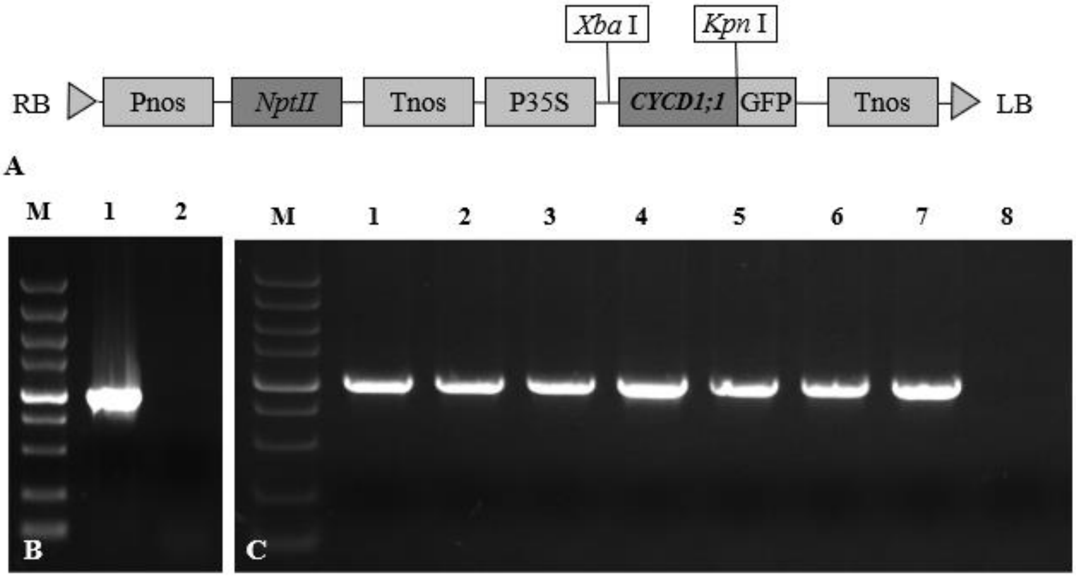

Fig. S1 The construction of pROKII-*PsnCYCD1;1-GFP* vector

A. Model of plant overexpression vector pROKII-*PsnCYCD1;1-GFP*. B. Cloning of *PsnCYCD1;1* gene. M, DNA marker DL 5000; 1, PCR product of *PsnCYCD1;1*; 2, negative control. C. PCR validation of pROKII-35S::*PsnCYCD1;1-GFP* transformants. M, DNA marker DL 5000; 1-7, PCR products of pROKII-*35S::PsnCYCD1;1-GFP* transformants; 8. negative control.

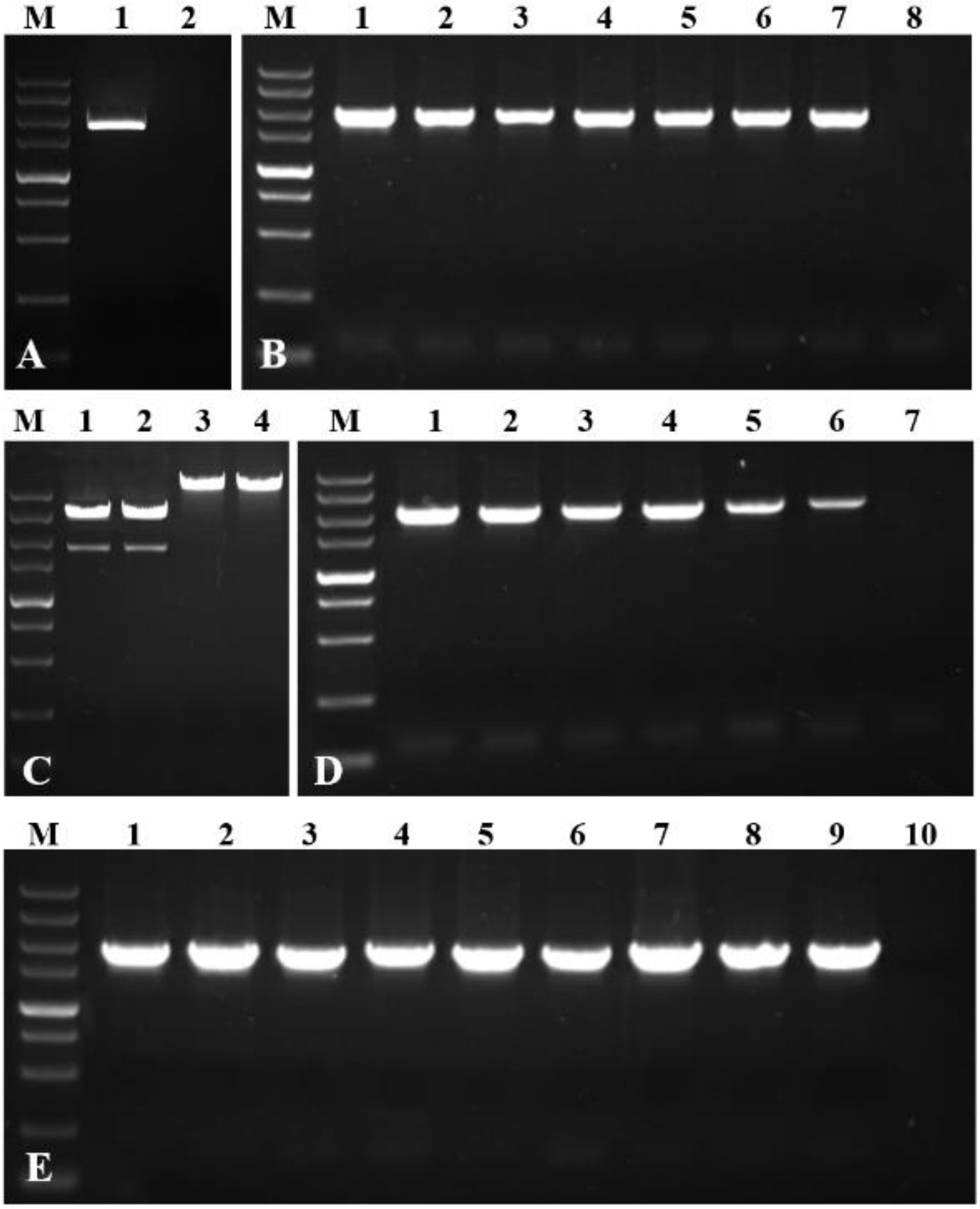

Fig. S2 The construction of pBI121-*PsnCYCD1;1-promoter-GUS* vector

A. Cloning of *PsnCYCD1;1* promoter. M, DNA marker DL 5000; 1, PCR product of *PsnCYCD1;1* promoter; 2, negative control. B. PCR detection of pEASY-*PsnCYCD1;1*-promoter transformants. M, DNA marker DL 5000; 1-7, PCR products of pEASY-*PsnCYCD1;1*-promoter transformants; 8. negative control. C. Double enzyme analysis of pEASY-*PsnCYCD1;1-promoter* and pBI121-*GUS*. M, DNA marker DL 5000; 1-2, digested results of pEASY-*PsnCYCD1;1*-promoter; 3-4, digested results of pBI121-*GUS* vectors. D. PCR detection of pBI121-*PsnCYCD1;1*-promoter transformants. M, DNA marker DL 5000; 1-6, PCR products of pBI121-*PsnCYCD1;1*-promoter transformants; 7. negative control. E. PCR validation of pBI121-*PsnCYCD1;1*-promoter transformants in GV3101. M, DNA marker DL 5000; 1-9, PCR products of pBI121-*PsnCYCD1;1*-promoter transformants in GV3101; 10. negative control.

```
+ TGATCCCGTG GTGGTGATCA GCACTAATTG ATTTCTGGAT TTTGCGAGAT AGCGAATAGG AGCGGAAACA -1871
- ACTAGGGCAC CACCACTAGT CGTGATTAAC TAAAGACCTA AAACGCTCTA TCGCTTATCC TCGCCTTTGT -1871
                                                                   AE-box
+ CGTTCGTGGA GACTTAGCAG ACTGATTTTA CAGTTTTCCT ATGTACTTTT GAATCCTTAC AAGTAGAAAC -1801
- GCAAGCACCT CTGAATCGTC TGACTAAAAT GTCAAAAGGA TACATGAAAA CTTAGGAATG TTCATCTTTG -1801
   MBS                  ABRE
+ AACTGTAACG CCTATGATGA TGGCTACGTG TACTGGTGTC TTAATTCGGA AAGGAACAGT AATTCCTGTC -1731
- TTGACATTGC GGATACTACT ACCGATGCAC ATGACCACAG AATTAAGCCT TTCCTTGTCA TTAAGGACAG -1731
                                      CAT-box      Box 4
+ TCTTCATGTC AGGAATTTAT TGTTGGGTTT CGTTATTTAG CCACTGGATT GATTAATGAG ATTCATTATT -1661
- AGAAGTACAG TCCTTAAATA ACAACCCAAA GCAATAAATC GGTGACCTAA CTAATTACTC TAAGTAATAA -1661
                                                 5UTR Py-rich stretch
+ GAATTTTACA GTAAAATCAT GGACAGATTC GTAATCTTCT TGAAGATTTT TCTACTCTTT TTCCCCTTTA -1591
- CTTAAAATGT CATTTTAGTA CCTGTCTAAG CATTAGAAGA ACTTCTAAAA AGATGAGAAA AAGGGGAAAT -1591

+ TATTATCATT TATCATTTCT CACCTATTAA ATCATGGACA GTAGTCAGTC CTCCTCTGCA TGCAAACAGC -1521
- ATAATAGTAA ATAGTAAAGA GTGGATAATT TAGTACCTGT CATCAGTCAG GAGGAGACGT ACGTTTGTCG -1521

+ TCATTAAAAA AATAATTTTG ATTGTAATGC ACATTAAATA TTTATTTTTC ATGATTTGAT ATGTATTATG -1451
- AGTAATTTTT TTATTAAAAC TAACATTACG TGTAATTTAT AAATAAAAAG TACTAAACTA TACATAATAC -1451
                        CAAT-box
+ TTTTTTTTCA TGGTATTTTT TATTTCAATA AAATAAAATA ATACATTTAA GTTAGATTTT TACAGATATT -1381
- AAAAAAAAGT ACCATAAAAA ATAAAGTTAT TTTATTTTAT TATGTAAATT CAATCTAAAA ATGTCTATAA -1381

+ GTACTGTATC GTAGTTTATG TATATTTTTT AAAAGTATTT TTTTATTTAA AAATATATTA AAATAATTTT -1311
- CATGACATAG CATCAAATAC ATATAAAAAA TTTTCATAAA AAAATAAATT TTTATATAAT TTTATTAAAA -1311
             ACE                  Box I
+ AATTTTTAAT ATTAACGTAT TAATAACTTT TCAAATAAAA TATATTTTAA AGATCACACA ACTTTATGAG -1241
- TTAAAAATTA TAATTGCATA ATTATTGAAA AGTTTATTTT ATATAAAATT TCTAGTGTGT TGAAATACTC -1241

+ GTGAAATAAC CTTACTGTTC CCTCTGTTAA AATCAAAGCT GTCTTGTCCT TTGTGTTTTC GAACTTGGTC -1171
- CACTTTATTG GAATGACAAG GGAGACAATT TTAGTTTCGA CAGAACAGGA AACACAAAAG CTTGAACCAG -1171
  TCT-motif                                                      GT1-motif
+ TCTTACACCA AAATTGGTAA GATGTTGGTC AATTAACTAC TTTTAATAAG TGCGTTTAAC AGGTTAACAT -1101
- AGAATGTGGT TTTAACCATT CTACAACCAG TTAATTGATG AAAATTATTC ACGCAAATTG TCCAATTGTA -1101
                          Box-W1                                      LTR
+ GTTAGAATAG GACTAAATTG CACATTTTAA AATACGAGTA CTTGATTGAA TTTTGCTCTT TTTTTCCGAA -1031
- CAATCTTATC CTGATTTAAC GTGTAAAATT TTATGCTCAT GAACTAACTT AAAACGAGAA AAAAAGGCTT -1031
   GC-motif  CCGTCC-box                         HD-Zip 3     circadian
+ AAACTCCCCC GTCCACTCAG TGTCGTGGTT ATTCGTTGGC TCGTAATCAT TCCCGCAACG AAATCTCCCA -961
- TTTGAGGGGG CAGGTGAGTC ACAGCACCAA TAAGCAACCG AGCATTAGTA AGGGCGTTGC TTTAGAGGGT -961

+ GGTTGCGCGC CGGACATGAA AGATCCCAGC GTCGAGGTTG TTGGAAGTAT TATAAAAAGT TCGATTCAGT -891
- CCAACGCGCG GCCTGTACTT TCTAGGGTCG CAGCTCCAAC AACCTTCATA ATATTTTTCA AGCTAAGTCA -891
                      CAAT-box
+ CACCGTCTTT CTTGAGACTG CAATTTGGCC TATTTCTAAG CATCTAACTT ATGGCATTTA TTGATGGGAC -821
- GTGGCAGAAA GAACTCTGAC GTTAAACCGG ATAAAGATTC GTAGATTGAA TACCGTAAAT AACTACCCTG -821
                                TGA-element  G-Box
+ AAATTAACCC AGTTTTGTAA ATTTTTGTAT AAACGACCAC GTTTGAAACA CAAGGATTGA GACTGTAAGG -751
- TTTAATTGGG TCAAAACATT TAAAAACATA TTTGCTGGTG CAAACTTTGT GTTCCTAACT CTGACATTCC -751
    CAAT-box
+ AAATCAATTT TACTGAAAAA ATAGAAAGGC TCAAACATAA AATGAAAACT TCGAAAAATC ATATCTATGA -681
- TTTAGTTAAA ATGACTTTTT TATCTTTCCG AGTTTGTATT TTACTTTTGA AGCTTTTTAG TATAGATACT -681
                                    MBS
+ AAAATTATAC ATTCAACAAA TAAAAATTAT TCAACTGTAA ATAATATATT TAAAATTAGC GGGAAAAGGA -611
- TTTTAATATG TAAGTTGTTT ATTTTTAATA AGTTGACATT TATTATATAA ATTTTAATCG CCCTTTTCCT -611
                                                                E2Fa

                                                               AT1-motif
+ TGTAGAGCGG GTTTAAGACT CTGAACAAAA TATTATTTTT TTAAAATTTA ATTTTTAAT TATTTTTAAT -541
- ACATCTCGCC CAAATTCTGA GACTTGTTTT ATAATAAAAA AATTTTAAAT TAAAAAATTA ATAAAAATTA -541

+ TGTTTTAATG TTATAATTTT TAAAATAAAA AATATATTAT TTTAATATAA AAAAAACACT TTAAAATAGT -471
- ACAAAATTAC AATATTAAAA ATTTTATTTT TTATATAATA AAATTATATT TTTTTTGTGA AATTTTATCA -471

+ ATTTGGAAAA ATCTCTACAG CGCTTTGATT AGATGTTAAA AATAAATGAA AAAAAAACAG GGAGTAGAGT -401
- TAAACCTTTT TAGAGATGTC GCGAAACTAA TCTACAATTT TTATTTACTT TTTTTTTGTC CCTCATCTCA -401
                      CAAT-box             Sp1
+ AGGGGCTAGG AAGGAGTAGG CAATGATAAA TCAGATGCCA CCCTACCAGG TCGCTTCACC TCCGTTCTTG -331
- TCCCCGATCC TTCCTCATCC GTTACTATTT AGTCTACGGT GGGATGGTCC AGCGAAGTGG AGGCAAGAAC -331
             CAAT-box                           TCT-motif
+ AAATGGAAAA TCAATTTTCC CGGTAAAACA AAGAACCCCA CACTGTCTTA CCTTCTCTCC CCTTTCCTTC -261
- TTTACCTTTT AGTTAAAAGG GCCATTTTGT TTCTTGGGGT GTGACAGAAT GGAAGAGAGG GGAAAGGAAG -261
   Box 4                 TATA-box                      HSE
+ ATTAATCTAC TTCCACTCTA AAATATATAA AAACAAAAAG AAAAGCAAAA AAAATTTGCA TCCCTTGATA -191
- TAATTAGATG AAGGTGAGAT TTTATATATT TTTGTTTTTC TTTTCGTTTT TTTTAAACGT AGGGAACTAT -191

+ TTTATATGGC TACAACACCA AACACTGATG GACATCCCTC CTCCATCTCT CTCCTAGCTC AACTTCGAAA -121
- AAATATACCG ATGTTGTGGT TTGTGACTAC CTGTAGGGAG GAGGTAGAGA GAGGATCGAG TTGAAGCTTT -121
                           circadian             GAG-motif
+ GCCTCTCTAG CTTAGCCCTT CACTACACAA GAGCATCCCG ATCACTACCC GGTGGAGTCT CCGGCCATCA -51
- CGGAGAGATC GAATCGGGAA GTGATGTGTT CTCGTAGGGC TAGTGATGGG CCACCTCAGA GGCCGGTAGT -51
                                                  -3-2-1 +1+2+3
+ TCGCGGCAAG ATGAGAGGAG AATGAGAAAC TATTATCATC AAAGGCTGCG ATG
- AGCGCCGTTC TACTCTCCTC TTACTCTTTG ATAATAGTAG TTTCCGACGC TAC
```

Fig. S3 Sequence and cis-elements in *PsnCYCD1;1* promoter

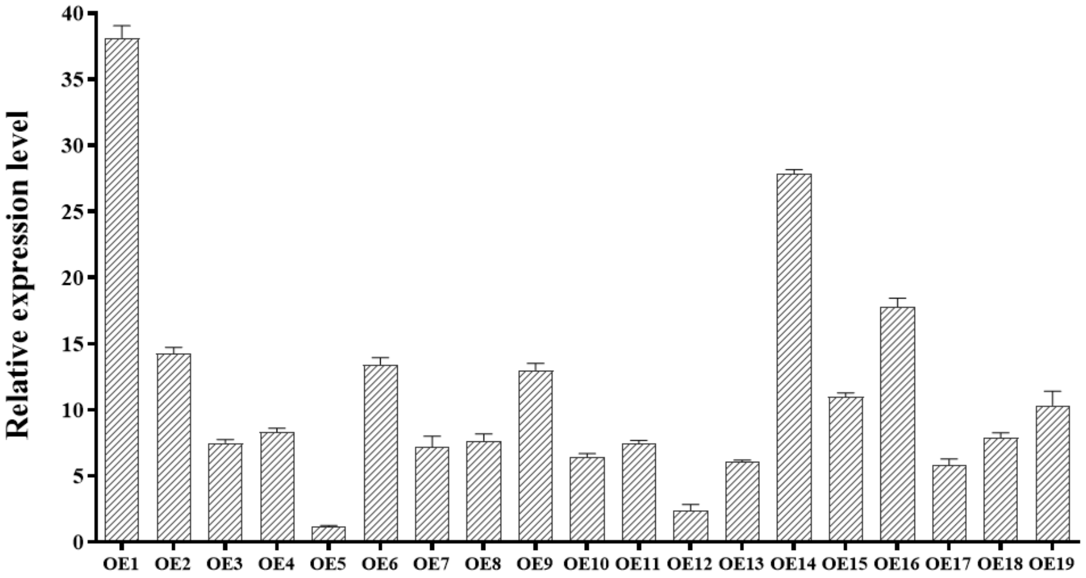

Fig. S4 The expression level of *PsnCYCD1;1* gene in transgenic tobacco

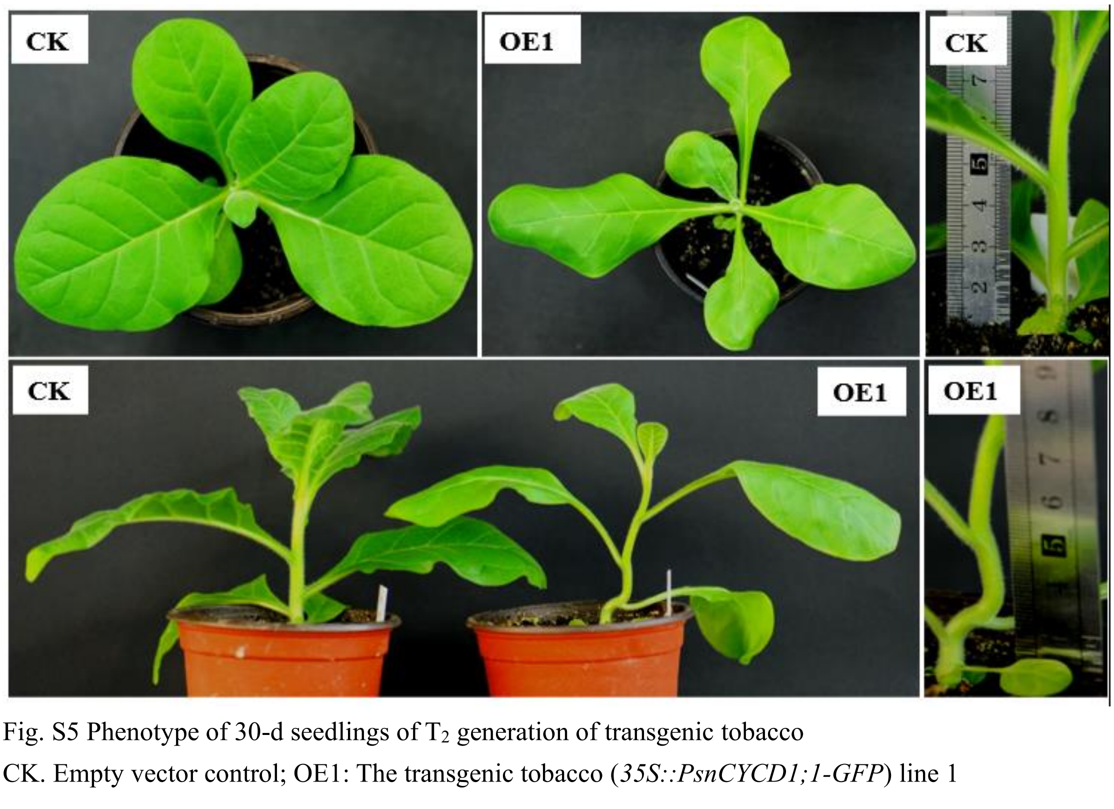

Fig. S5 Phenotype of 30-d seedlings of T_2 generation of transgenic tobacco

CK. Empty vector control; OE1: The transgenic tobacco (*35S::PsnCYCD1;1-GFP*) line 1

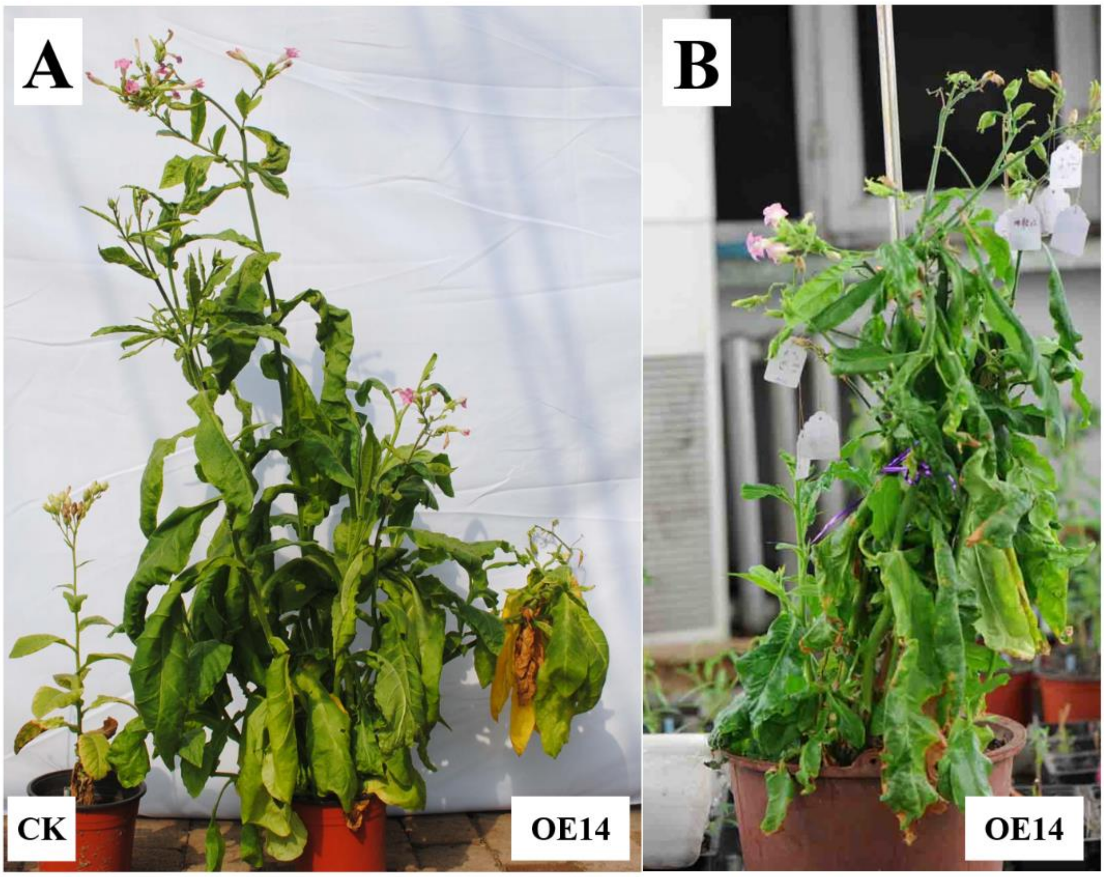

Fig. S6 Special transgenic lines (OE14) that maintain sustained growth

CK. Empty vector control; OE14: The transgenic tobacco (*35S::PsnCYCD1;1-GFP*) line 14

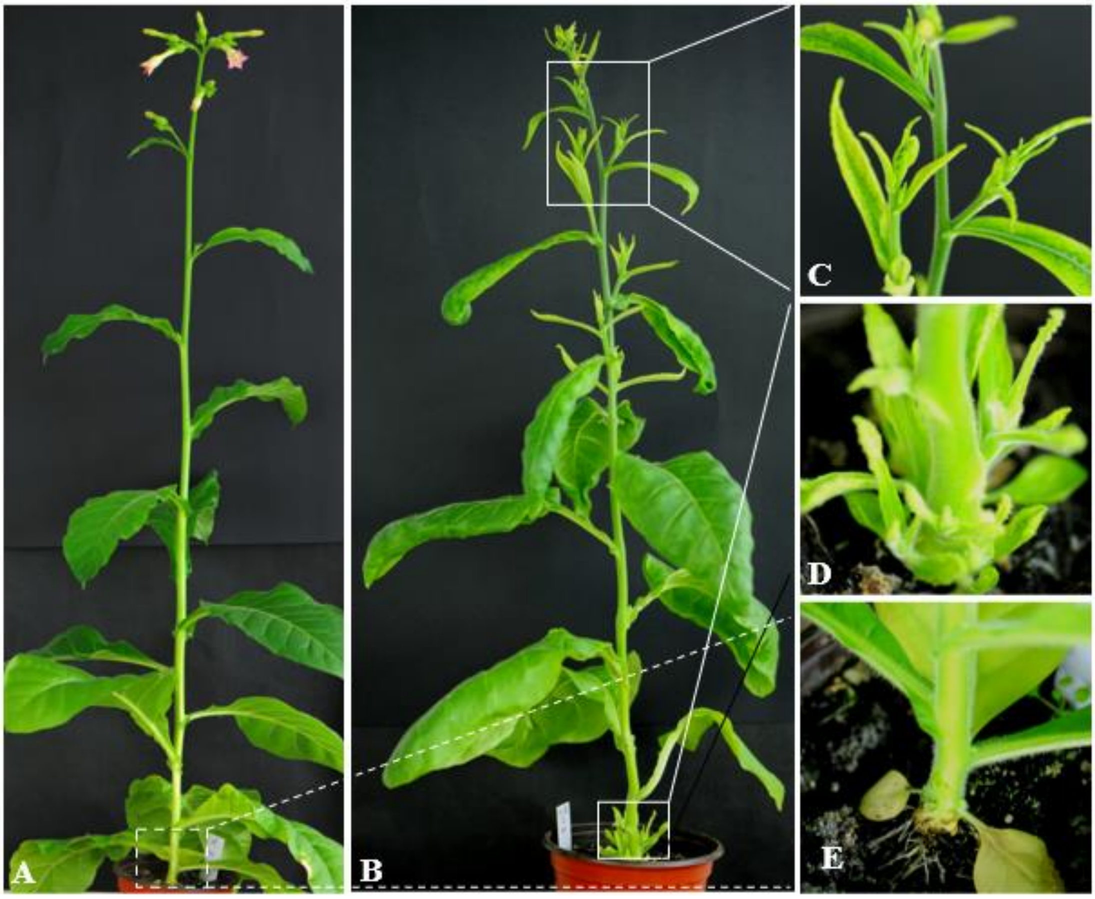

Fig. S7 Phenotype of 60-d seedlings of T_2 generation of transgenic tobacco

A 60 d old control tobacco； B 60-d transgenic line 2； C, D, E Close-up of parts of A or B

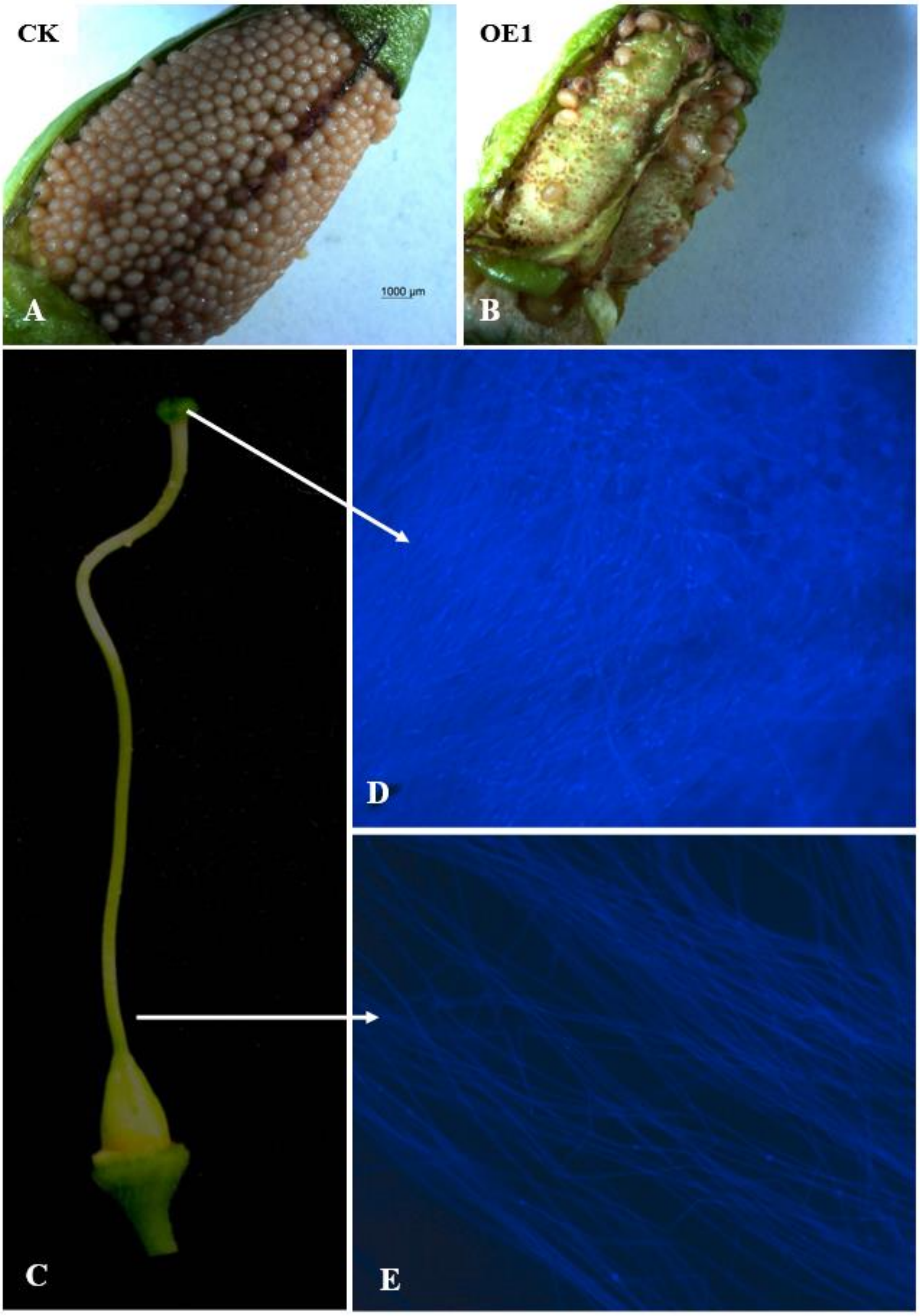

Fig. S8 Fertility analysis of transgenic tobacco

A and B. Microscopic observation of seeds in the ovary 10 days after pollination with own pollens.
C. Schematic diagram of pistil of transgenic tobacco. D. Staining of pollen tubes on stigma using water-soluble aniline blue staining. E. Staining of pollen tubes on base of style using water-soluble aniline blue staining.

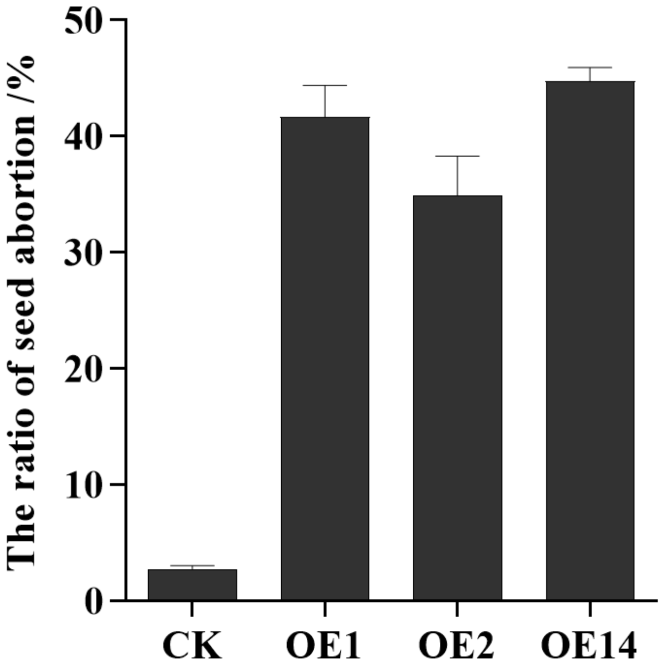

Fig. S9 Comparative analysis abortion rates between control and transgenic tobacco
OE1/2/14: Three individual transgenic lines.

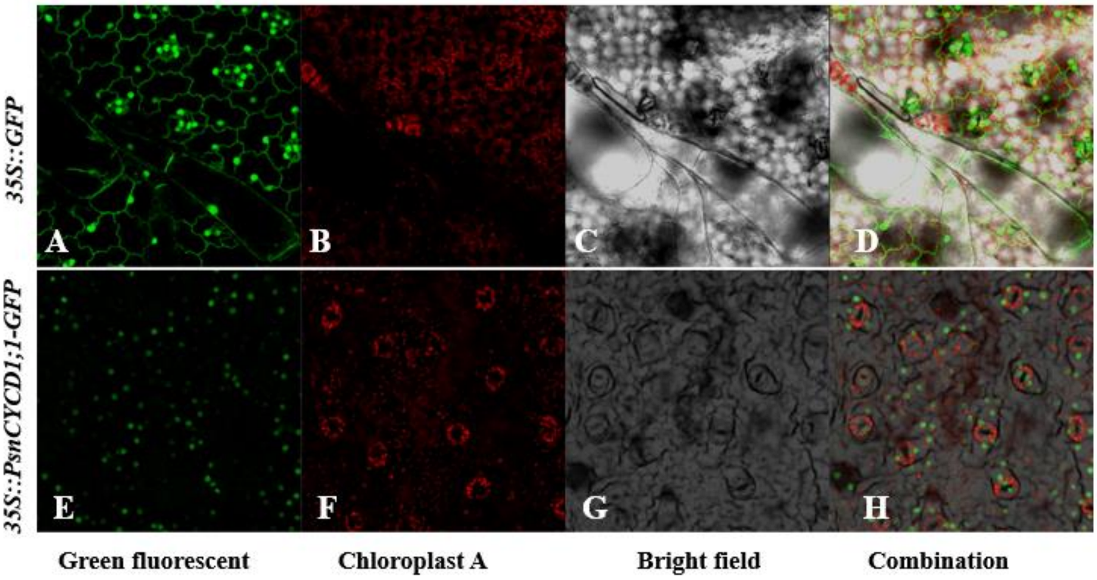

Fig. S10 GFP observation of tobacco leaf lower epidermis
35::GFP: pROKII-*35::GFP* empty vector control; *35S::PsnCYCD1;1-GFP*: fusion vector of *PsnCYCD1;1* and *GFP*; Green fluorescent: green fluorescence signal; Chloroplast A: Chloroplast autofluorescence; Bright field: white light; Combination: Combined signals of different fluorescence.

Supplementary Tables

Table S1 The primer sequences used in this study

Name	Sequence (5'--3')	Usage
PsnCYCD1;1-GFP-F	ATCGTCGACGAGATGGCCTACTCTGATTGCTTATCAG	Plant expression vector
PsnCYCD1;1-GFP-R	ATCCCATGGCCTCGGAATTTCCTTTGTCA	
PsnCYCD1;1-Pro-F	ATCAAGCTTCTGTAACGCCTATGATGATGGCTAC	Promoter cloning
PsnCYCD1;1-Pro-R	ATCGGTACCCGCAGCCTTTGATGATAATAGTTTCTC	
GUS-F	GGGCGAACAGTTCCTGATTA	
GUS-R	CGAAATATTCCCGTGCACTT	
PsnCYCD1;1-RT-F	CCTCTGGTTCCTTCTCTATTGG	qRT-PCR
PsnCYCD1;1-RT-R	CAGAAACCCAGTATAGGCTCC	
NtActin-RT-F	TGTGTTGGACTCTGGTGATG	
NtActin-RT-R	CGCTCGGTAAGGATCTTCATC	
NtCYCD1-F	GTGCTTCTCCGACTTACTCTG	
NtCYCD1-R	TCTCGAAATAGTCAAATCCAGGG	
NtCYCD2-F	TGGAATACAAGCCTTCTGAGATAG	
NtCYCD2-R	GATGGAATTGGTGCACTTGG	
NtCYCD3-F	TTTGTTTTGGGAAGATGAAGAGC	
NtCYCD3-R	GCAGTCAAAGCAGAGAAACC	
NtCYCD5-F	ATCGCCAACCCTATACTCAATG	
NtCYCD5-R	TGTCCGTGTAGCAAGAAACC	
NtCYCD6-F	AGCTAATGCGGGATGTTACAG	
NtCYCD6-R	AACCACTCAATCTTCGCCTC	
NtCYCD7-F	GCTTGAATTACAGATGGAAGGTG	
NtCYCD7-R	AGATGGGTAATTTGAGGGACTTG	
NtCDKA1-F	CTGGTGACTCCGAGATTGATG	
NtCDKA1-R	CCAAGCGGAGCATTTTATCAAG	
NtCDKB1-F	GATTGCTGATTTGGGTCTTGG	
NtCDKB1-R	AGAGTCACCAGGAAATAAGGC	
NtE2F-F	GGGAGATGTGGAAAATTTGACG	
NtE2F-R	CCATGTGGAGCTTTGATTGC	
NtEXP10-F	AGGATCAAGAACTGGTTGGC	
NtEXP10-R	TCCAAAAGACCAATGAGCAGG	
NtSTM-F	CCTCGTCTCTTGTCTGCTTATG	
NtSTM-R	CCACTGTTACGGCCTATTGTAG	

NtKNAT1-F	GCTCATCCTCAGTGTTCTAACC
NtKNAT1-R	GATCAAGTTCTGGGTCCTTAGC
NtAS1-F	AGATTGTGAAAGAGCGGAGTG
NtAS1-R	TGACGGTTGAAGTAGCAGTG
NtAS2-F	GGCCGAACTGCTAAAAGATTG
NtAS2-R	CGTATTTTCCCTCGTCTACTGG
